# 蛋白激酶CK2与人类恶性肿瘤

**DOI:** 10.3779/j.issn.1009-3419.2012.07.09

**Published:** 2012-07-20

**Authors:** 轶轩 黄, 少华 周, 志龙 赵

**Affiliations:** 116001 大连，大连大学附属中山医院胸外科 Department of Thoracic Surgery, Zhongshan Hospital, Affiliated to Dalian University, Dalian 116001, China

恶性肿瘤严重威胁人类健康和生命，影响社会经济的发展。随着研究的深入，恶性肿瘤发生发展机制、生物学行为调控机理不断被揭示，新的药物靶点陆续被发现，如蛋白激酶CK2（casein kinase 2或Ⅱ）。在肺癌、消化系统肿瘤、头颈部鳞状细胞癌、男性泌尿生殖系肿瘤、女性生殖系统肿瘤、恶性黑色素瘤、血液系统恶性肿瘤中，CK2的表达水平和活性明显增高，不仅与肿瘤细胞扩散能力、癌症预后相关，还反映其病理生理学特征^[1-5]^。近年CK2与恶性肿瘤的相关研究较多，针对CK2的肿瘤靶向治疗策略越发清晰，相关抑制剂大量涌现，本文就此综述如下。

## 1 CK2的结构与功能特点

CK2最早被称为酪蛋白激酶Ⅱ，是一种信使非依赖性、丝/苏氨酸激酶，在真核细胞中普遍存在，进化过程高度保守。全酶呈四聚体，核心为调节亚基β二聚体、结合催化亚基α和/或α'二聚体（即：ααββ、αα'ββ或α'α'ββ）。人类CK2催化亚基还有一个剪接异构体。同CK2α相比，CK2α'与CK2β的亲和力低12倍，并不易被焓驱动。CK2α/CK2β连接的关键部位-β4/β5环在CK2α'为开放构象，而CK2α仅在同CK2β组装后开放。CK2α'的β4/β5环稳定开放有两个因素，N末端β折叠被额外的β链延长和色氨酸残基充填于β4/β5环和αC螺旋之间的、保守的疏水凹槽。此外，CK2α'的域间铰链区为全功能构造，而非结合的CK2α常为非生产性铰链构造，并仅通过与CK2β结合才能被克服。在功能临界区域，CK2α'更加不依赖于CK2β来获得完全的功能构象^[6]^。

某些时候，α/α'亚基可单独存在于细胞中发挥催化作用。针对胚胎发育的研究显示，缺少CK2α时尽管有CK2α'存在，仍会出现CK2酶活性明显下降；CK2α-/-的胚胎会出现很多重大畸形，并死于妊娠中期。同时，CK2β与CK2α一样对于正常胚胎发育也是必需的^[7]^；敲除CK2α'的基因可使动物精子细胞发生染色质缺陷而死亡^[8]^。在不同组织和肿瘤中CK2催化和调节亚基表达不平衡，每个亚基具备独立催化功能。利用活细胞成像进行定位研究^[9]^发现，CK2催化和调节亚基广泛共定位，同时具有独立的流动性。

CK2的一个重要特征是利用三磷酸腺苷（adenosine triphosphate, ATP）或三磷酸鸟苷（guanosine triohosphte, GTP）作为磷酸基团供体，具有众多磷酸化蛋白底物，达300余种，而且新发现的底物不断增加。这些底物涉及信号传导、基因复制、转录、加工和翻译，在细胞存活、生长、增殖、分化、凋亡和老化过程中发挥作用。因此，CK2被认为具有重要生命功能而处于细胞生长调控的中心地位^[10, 11]^。其作用机制非常复杂，调控网络仍未完全明了。但是，已经明确的是选择性下调CK2的表达和活性可以阻止肿瘤细胞生长^[12]^。

## 2 CK2与恶性肿瘤的关系

### 2.1 血液系统肿瘤

Kim等^[5]^发现在具有正常核型的急性髓细胞样白血病患者中CK2α是预后的独立影响因子。虽然CK2α表达与具体的病理生理学影响没有被评价，但作为不良的预后因素，CK2α的表达水平可用来评估成人患者的预后风险、复发可能性，并可用来指导治疗。值得注意的是，CK2抑制剂，如芹菜素，可选择性诱导CK2α异常表达的白血病细胞出现凋亡，而不会影响正常造血干细胞。CK2抑制剂诱导白血病细胞凋亡的能力与细胞中CK2表达水平密切相关，CK2α表达水平越高，抑制剂的敏感性越高。

在慢性淋巴细胞白血病细胞的存活方面，CK2发挥重要功能，CK2抑制剂可介导蛋白激酶C失活，引起慢性淋巴细胞白血病细胞死亡，作用位点在磷脂酰肌醇3激酶的下游，与人10号染色体缺失的磷酸酶及张力蛋白同源物的活性增加相关^[13]^。针对多发骨髓瘤细胞的体外实验^[14]^证实，CK2抑制剂芹菜素具有抑制增殖和诱导凋亡的效果。

此外，在多发性骨髓瘤、急性成淋巴细胞性白血病、慢性骨髓增生性肿瘤等血液肿瘤发病机制中，CK2也发挥重要作用。在血细胞生成相关的信号通路中，CK2调节并加强生化级联反应，这对肿瘤生长、增殖、拮抗传统和新的细胞毒药物都必不可少^[15]^。

### 2.2 实体肿瘤

#### 2.2.1 男性泌尿生殖系肿瘤

Laramas等^[16]^发现CK2α核定位与前列腺癌的病理分级、临床分期、局部和淋巴侵袭相关，是前列腺癌的一项恶性指标。前列腺癌细胞中CK2含量明显增高。无论对雄激素敏感与否，CK2含量降低，可增强肿瘤坏死因子相关凋亡诱导配体（TRAIL）诱导的细胞凋亡作用。CK2的短暂表达，即可使前列腺癌细胞逃脱TRAIL诱导的凋亡^[17]^。CK2活性增强除了可阻断TNFα诱导的凋亡^[18]^，还可下调前列腺特异性同源框基因NKX3.1的表达，使前列腺上皮细胞向癌细胞转化。CK2抑制剂能增强NKX3.1的抑癌作用，诱导前列腺癌细胞凋亡，防止正常前列腺上皮细胞癌变^[19]^。

CK2抑制剂或小干扰RNA（siRNA）能够特异性抑制前列腺癌细胞，而对良性细胞没有作用^[20]^。Götz等^[21]^发现可穿透细胞的人蛋白激酶CK2选择性抑制剂能有效诱导前列腺癌细胞凋亡。Trembley等^[22]^证明了全身给予针对CK2α和α'亚基的反义寡核苷酸能使小鼠前列腺癌原位移植瘤缩小。

CK2α、环氧化酶2（cyclooxygenase-2, COX-2）以及磷酸化蛋白激酶B（Akt）在晚期、侵润性、或原位膀胱癌中存在高表达。同时，以COX-2依赖或不依赖方式，CK2α-Akt/尿激酶型纤维蛋白酶原激活剂（urokinase-type plasminogen activator, uPA）信号激活，进而促进膀胱上皮癌生长。再者，对于膀胱上皮癌治疗来说，不仅是COX2，CK2α也可作为COX2抑制剂的靶点^[23]^。

#### 2.2.2 女性生殖系统肿瘤

同正常子宫内膜组织相比，子宫内膜癌组织中CK2β表达率和强度都明显升高，并同组织学类型、分级、分期、细胞增殖以及凋亡指数相关，还与cyclin D1、PTEN、AKT、β-连环蛋白（β-catenin）、FLIP（Flice-like inhibitory protein）等的表达相关。利用短发夹RNA（shRNA）下调CK2β，促使癌细胞集落形成受阻，进一步证明了CK2β同子宫内膜癌的增殖相关^[24]^。最新研究^[25]^发现，CK2抑制剂-醌茜素可阻止子宫内膜组织的血管萌生，并且作用呈剂量依存性。值得注意的是，CK2活性的下调，没有影响到α、α'和β 3个亚基的表达，提示抑制CK2活性影响血管再生，可能成为子宫内膜病变治疗的新策略。

针对宫颈癌细胞的研究显示CK2介导细胞存活，涉及对六磷酸肌醇激酶-2（IP6K2）去稳定化。生理上CK2可磷酸化IP6K2中PEST序列的S347和S356氨基酸残基，这些残基正是泛素化的常见位点^[26]^。不同化学结构的CK2抑制剂的促凋亡作用，都可因为IP6K2的缺乏而明显下降。因此，针对CK2调控IP6K2的靶点进行阻滞，可能是有效的靶向治疗策略。

#### 2.2.3 乳腺癌

利用高通量密度测量法和数字化组织芯片成像，对1, 000例乳腺癌组织进行免疫组化染色，检测33个生物学标记物的临床相关性。结果显示CK2α是癌转移和预后的影响因子，其高表达预示着不良预后。因此CK2以及关键的下游因子可能是特异性治疗的潜在靶点^[27]^。

在炎性乳腺癌生成过程中，促血管生成因子以及促炎症细胞因子发挥重要作用。其中，白介素-6（IL-6）同癌细胞增殖和生存相关，并促进血管发生、炎症过程和转移。在炎性乳癌细胞和动物模型中，利用siRNAs以及小分子抑制剂CX-4945，通过下调CK2表达可抑制IL-6分泌，产生抗癌作用。1例炎性乳腺癌患者应用CX-4945后，其血浆中IL-6水平明显下降。因此，在IL-6相关癌症或其它疾病中CX-4945等CK2抑制剂可能具有治疗价值^[28]^。另据报道^[29]^口服CX4945可使癌细胞增殖周期停止，选择性诱导凋亡发生，从而抑制癌细胞增殖。对正常细胞则无此作用。此外，还可阻止血管生成信号传导，抑制血管内皮细胞生成。这些作用的发挥有赖于CK2表达的抑制。

在人乳腺癌、结肠癌以及人胚肾细胞中，利用SC-791选择性抑制COX-2，或利用2-二甲氨基-4, 5, 6, 7-四溴-1H-苯并咪唑（DMAT）抑制CK2，都可下调COX-2表达以及细胞生存力。另一方面，CK2α异常表达可以通过Wnt/β-catenin途径的活化来促进COX-2上调。无论过表达CK2α、β-catenin或COX-2，还是介质中添加前列腺素E2，都可有效克服DMAT或显性失活CK2α引起的细胞生存抑制。因此，在癌症发生中，CK2通过上调COX-2表达和前列腺素E2产生而发挥作用^[30]^。

#### 2.2.4 头颈部肿瘤

头颈部鳞癌是世界第7常见癌，在过去的40年，其生存未有明显改善，因此需要新的治疗靶点。研究^[31]^表明正常喉粘膜、喉癌前病变、喉癌组织中，CK2表达水平呈递增趋势。CK2 siRNA在体外^[32]^、体内^[33]^均可抑制喉癌细胞生长。胚胎形态发生的主要调节因子Twist是上皮-间质细胞转化以及癌症转移的主要调节因子。IL-6可增加Twist表达。血清IL-6水平和癌组织Twist表达，与头颈部鳞癌不良预后相关。CK2可磷酸化Twist残基S18、S20，阻止其降解，从而提高了细胞存活力^[34]^。CK2还可调节核转录因子（NF）-κB活化、P53/p63以及下游基因的表达。无论体内还是体外下调人头颈部鳞癌中的CK2即可产生抗癌作用，并增加顺铂敏感性^[35]^。因此，CK2可能是头颈部鳞癌的治疗靶点。

#### 2.2.5 神经胶质瘤

多形性胶质母细胞瘤细胞中存在高水平的CK2，可以抵抗肿瘤坏死因子α诱导的凋亡。CK2抑制剂-DRB或芹菜素，可下调胶质瘤细胞的生存力，阻止TNFα介导的NF-κB活化，增加细胞敏感性。更重要的是，CK2抑制剂可激活野生型*p53*，而不是突变型*p53*。p53功能激活涉及增强的转录活性和DNA结合能力，以及同细胞周期进展和凋亡相关的*p53*靶基因表达的增强。再者，CK2抑制剂可降低端粒酶活性，以p53依赖方式促使老化的发生。CK2抑制剂还可降低鼠双微基因2（MDM2）-p53的结合以及p53泛素化，从而提高p53水平，同时抑制组蛋白去乙酰化酶1（SIRT1）的活性^[36]^。

#### 2.2.6 消化系统肿瘤

胃癌是常见的恶性肿瘤。对104例胃癌和癌旁正常胃组织进行免疫组化染色显示，细胞核CK2β高表达与癌浸润深度明显相关；CK2β高表达患者的预后明显不良。多因素分析显示细胞核CK2β高表达和分期是胃癌预后的独立影响因子（*P*值分别为0.003, 6、0.000, 5）。因此，CK2β高表达可被看作是胃癌不良预后的标记物^[37]^。

同结直肠腺瘤相比，结直肠癌细胞核中CK2α表达明显增强。利用siRNA或抑制剂-布洛芬，抑制CK2α活性，可阻止癌细胞增殖，使其停滞于G_0_/G_1_期，诱导细胞老化，p53/p21表达增加。同时，敲除CK2α后细胞运动和侵袭能力下降。下调CK2α可激活β-catenin，降低波形蛋白和转录因子snail和smad2/3的表达，并增加E-钙粘蛋白表达，提示CK2α调节癌细胞上皮-间质转化过程。研究^[38]^认为CK2α在结直肠癌的发生发展过程中发挥重要作用，抑制CK2α可能是人结直肠癌治疗的新策略。

同样，利用免疫组化、实时定量PCR、Western印迹方法，检测245例结直肠癌组织中CK2α的表达。结果显示，胞核中CK2α高表达与癌浸润深度、淋巴结受累、分期、分化程度、以及嗜神经侵袭明显相关；CK2α高表达患者预后明显不良。多因素分析证实CK2α是结直肠癌预后的独立影响因子。利用siRNA下调CK2α可抑制癌细胞生长^[39]^。提示CK2α不仅可能是结直肠癌预后的标记物，还可能是药物治疗靶点。CK2特异性抑制剂-四溴-2-苯并三唑可增强放射线诱导的癌细胞死亡^[40]^。

高表达的磷脂酶D可防止细胞周期停滞和凋亡发生。在人结直肠癌细胞中其亚型-磷脂酶D2通过泛素依赖方式加速CK2β降解，并影响CK2的活性；这一调节作用依赖于磷脂酶D2的C末端域，而不是其催化活性^[41]^。

由于缺乏有效治疗手段，胰腺癌5年生存率为3%，中位生存期不足6个月，现有靶向药物治疗胰腺癌无论是单药还是联合用药均无明显效果。Giroux等^[42]^对人类着丝粒进行系统筛查，寻找影响胰腺细胞生存的激酶，确定了56个激酶可能是胰腺癌治疗靶点。在临床前期的体内外研究中，联合抑制激酶PAK7、MAP3K7和CK2，具有诱导癌细胞凋亡的累积效果，并对胰腺癌细胞具有特异性。

利用siRNA下调CK2α或α'，联合应用吉西他滨，可导致胰腺癌细胞发生凋亡和坏死性死亡。下调细胞内CK2α，有丝分裂原激活蛋白激酶4/应激活化蛋白激酶磷酸化并活化；而CK2α'下调主要对PI3K/Akt途径发挥作用。CK2α'下调可对吉西他滨诱导细胞死亡施加更大影响^[43]^。CK2小分子抑制剂CX-4945可有效抑制人胰腺癌小鼠移植瘤的生长^[29]^。这些都说明CK2有望成为胰腺癌新的治疗靶点。

高表达的拓扑异构酶Ⅱα同肝癌进展和化疗抵抗相关，组蛋白脱乙酰基酶抑制剂可使肝癌细胞中拓扑异构酶Ⅱα发生选择性降解，可能是肝癌治疗的新策略。Chen等^[44]^发现，组蛋白脱乙酰基酶抑制剂促进乙酰化组蛋白H3同CK2α基因启动子结合，使CK2α转录活化，从而增加其表达。CK2α磷酸化拓扑异构酶Ⅱα，使其同信号小体亚基5结合，从而使拓扑异构酶Ⅱα发生泛素依赖的降解，CK2α在这一过程中发挥着启动激酶的作用。

#### 2.2.7 非小细胞肺癌

同正常肺组织相比，非小细胞肺癌中CK2表达和活性明显增高，mRNA表达水平与激酶活性基本平行。此外，癌组织中CK2α表达高，而癌旁正常组织中CK2α'表达更高；同腺癌相比，鳞癌中CK2β表达明显增高^[45]^。另有学者^[46]^证实，CKα是肺鳞癌预后的独立影响因子，提示CK2α或β可能是理想的非小细胞肺癌生物学标志物。相比于腺癌，现有靶向药物对鳞癌缺乏疗效；通过抑制CK2可能为鳞癌靶向治疗打开一扇窗。

肿瘤抑制因子-早幼粒细胞白血病蛋白（promyelocytic leukemia, PML）调控生长抑制、诱导凋亡、细胞老化，在实体和血液肿瘤中常发生缺失，并同肿瘤进展相关。同非小细胞肺癌相比，小细胞肺癌细胞核中，PML缺失更明显，其缺失发生于基因转录后^[47, 48]^。另外，在肺癌组织中，PML缺失同p53、P16INK4A^[49]^的表达相关。Scaglioni等^[50]^发现非小细胞肺癌中PML同CK2α的表达成反比；通过磷酸化PML的Ser517，CK2促使泛素-蛋白酶体降解PML，从而导致其肿瘤抑制活性丧失。因此，小细胞肺癌中PML下调，可能是CK2α介导的降解所致。值得注意的是，CK2α活性下调70%，PML表达即可增高2倍，并在体内外抑制癌细胞生长，此作用有赖于PML活性的恢复；同时还发现CK-2α和β亚基在肺癌中表达增高，并同PML低表达相关。因此，对于CK2活性异常增高和PML蛋白丢失肿瘤尤其是小细胞肺癌，特异性CK2抑制剂可能具有疗效。Hung等^[51]^发现，CK2α无内含子基因在非小细胞肺癌中出现扩增，其特异性siRNA可明显增加肺癌中PML蛋白水平，因此可能是原癌基因。

CIGB-300是一种促凋亡肽药物，能阻止CK2的磷酸化作用，体内外都具有抗肺癌活性。CIGB-300与NSCLC细胞短时间孵育（45 min），利用蛋白组学方法确定了137种蛋白的表达下降2倍以上。CIGB-300引发表达水平改变的蛋白涉及核糖体生物合成、细胞生长增殖、凋亡、转移和药物抵抗，提示CIGB-300具有抗癌药物的潜质^[52]^。Lin等^[53]^发现针对非小细胞肺癌CK2抑制剂具有放射增敏潜能。

## 3 蛋白激酶CK2的抑制剂

按照作用原理，CK2抑制剂可分为4类^[54]^：①磷酸受体底物类似物，通过与磷酸受体底物竟争性结合CK2，而抑制其活性；②磷酸供体底物类似物，分为ATP和GTP竞争性抑制剂两类，前者研究较多，GTP竞争性抑制剂如酪氨酸磷酸化抑制剂AG114、AG213、AG1109、AG1478等对CK2亦有较强抑制作用；③破坏活化环与N-末端区段间相互作用的化合物；④能阻断与β亚基结合的化合物，CK2催化核心同其它人类蛋白激酶具有很高的同源性，其ATP结合位点可作为多数小分子抑制剂的靶点，这些化合物类似于腺嘌呤，通过疏水性相互作用以及氢键结合等复杂网络靶向ATP结合袋。因此，最初甄选的CK2抑制剂是ATP竞争性抑制剂。

### 3.1 ATP竞争性抑制剂

包括卤代化合物-四溴苯并咪唑衍生物、浓聚多酚衍生物、吲哚喹唑啉类化合物等，为数众多。在低微摩尔级即可产生效果，并有较高特异性。有些或许因脱靶至其它存在相似ATP结合位点的ATP结合蛋白而表现不同的生理意义^[55, 56]^。吡啶卡巴唑和benzopyridoindole衍生物-新的ATP竞争性抑制剂，显示了抗人恶性胶质瘤小鼠移植瘤的活性^[57]^。

研究发现CX-4945具有广谱抗癌的结构特点^[58]^。与EGFR酪氨酸激酶抑制剂erlotinib联合使用，在体内外具有协同抗非小细胞肺癌（non-small cell lung cancer, NSCLC）作用^[59]^。CX-4945有效抑制CK2活性的同时，可增加吉西他滨和铂类化疗药物的疗效，有望成为化疗增敏剂^[60]^。它还是第一个进入临床试验的口服型CK2生物相容性小分子抑制剂，从而使靶向治疗癌症进入一个全新阶段。

小分子CK2抑制剂-苏木因，具有细胞穿透性和有效的肿瘤细胞抑制作用^[61]^。天然化合物-试卤灵是CK2高效抑制剂，并具有高选择性。在52个激酶中只有CK2被其抑制，并可明显抑制肿瘤细胞生长^[62]^。

### 3.2 非ATP竞争性抑制剂

由于CK2存在嗜酸性底物特异性，利用多肽同CK2底物的酸性磷酸受体结合，从而阻断CK2催化性磷酸化，继而诞生了第二代抑制剂^[54, 63]^。在人体试验^[64]^中初步证实对宫颈癌具有治疗效果以及安全性。有别于前两种方式，一种独特类型的无机化合物-多金属氧酸盐被确认同CK2α外部位点结合，强效抑制CK2的催化活性^[65]^。

CK2α亚基铰链/αD区、p-环、β4β5环具有异常灵活性，这些构造可变区域之间并无相关性。人类酶铰链/αD区构造可变性在所有蛋白酶中是独一无二的，这一特点可被用来开发更具选择性的ATP竞争性抑制剂。确定配体结合第二位点的独特模式，也能为针对α和β亚基间相互作用的、非ATP竞争性抑制剂的设计提供线索^[66]^。

选择性CK2抑制剂-TF具有细胞穿透性，通过诱导凋亡表现出明显的抗癌活性^[21]^。白藜芦醇-蛋白酪氨酸激酶抑制剂，可抑制人乳腺癌细胞增殖，其有效IC_50_值为30 μmol/L-45 μmol/L。该作用有赖于对p38MAPK/CK2信号通路的启动，抑制雌激素受体α mRNA和蛋白表达。对于抗他莫昔芬的乳腺癌细胞来说这种效果更加持久。因此，白藜芦醇有望成为激素抵抗乳腺癌的辅助药物^[67]^。

近年来，随着免疫学和分子生物学发展，CK2单克隆抗体和反义核酸已受到重视，并可制备高度特异性的单克隆抗体和特异性载体，导向目的细胞，提高药物疗效，减少毒性；通过反义核酸技术有效调节*CK2*基因表达，同时也显示了对肿瘤的治疗作用^[22, 39, 50]^。

## 4 展望

在细胞生长过程中CK2的功能复杂又重要。不需要完全抑制CK2，仅部分降低其活性就可抑制癌基因作用^[50, 68]^。现有研究^[69]^从不同方面揭示，CK2具有艾滋病、肿瘤等疾病药物治疗靶标的潜能，其特异性抑制剂的研究越来越多。有学者^[12]^认为CK2是治疗肿瘤的多目标靶点。恶性肿瘤生物学特性存在差异，分子调控机制不尽相同。因此，明确不同肿瘤的独特信号传导通路，开展有针对性的药物研发势在必行，这也符合基于分子水平的个体化肿瘤治疗原则。
